# A multiple T cell epitope comprising DNA vaccine boosts the protective efficacy of Bacillus Calmette–Guérin (BCG) against *Mycobacterium tuberculosis*

**DOI:** 10.1186/s12879-020-05372-1

**Published:** 2020-09-17

**Authors:** Sudeep Kumar Maurya, Mohammad Aqdas, Deepjyoti Kumar Das, Sanpreet Singh, Sajid Nadeem, Gurpreet Kaur, Javed Naim Agrewala

**Affiliations:** 1 CSIR-Institute of Microbial Technology, Chandigarh, 160036 India; 2 Present Address: Indian Institute of Technology, Rupnagar, 140001 India

**Keywords:** T cells, Epitopes, DNA vaccine, BCG, Tuberculosis

## Abstract

**Background:**

Approximately 80% - 90% of individuals infected with latent *Mycobacterium tuberculosis (Mtb)* remain protected throughout their life-span. The release of unique, latent-phase antigens are known to have a protective role in the immune response against *Mtb*. Although the BCG vaccine has been administered for nine decades to provide immunity against *Mtb*, the number of TB cases continues to rise, thereby raising doubts on BCG vaccine efficacy. The shortcomings of BCG have been associated with inadequate processing and presentation of its antigens, an inability to optimally activate T cells against *Mtb*, and generation of regulatory T cells. Furthermore, BCG vaccination lacks the ability to eliminate latent *Mtb* infection. With these facts in mind, we selected six immunodominant CD4 and CD8 T cell epitopes of *Mtb* expressed during latent, acute, and chronic stages of infection and engineered a multi-epitope-based DNA vaccine (C6).

**Result:**

BALB/c mice vaccinated with the C6 construct along with a BCG vaccine exhibited an expansion of both CD4 and CD8 T cell memory populations and augmented IFN-γ and TNF-α cytokine release. Furthermore, enhancement of dendritic cell and macrophage activation was noted. Consequently, illustrating the elicitation of immunity that helps in the protection against *Mtb* infection; which was evident by a significant reduction in the *Mtb* burden in the lungs and spleen of C6 + BCG administered animals.

**Conclusion:**

Overall, the results suggest that a C6 + BCG vaccination approach may serve as an effective vaccination strategy in future attempts to control TB.

## Background

*Mycobacterium tuberculosis* (*Mtb*) kills 1.5 million people annually [1]. Furthermore, the increasing frequency of *Mtb* cases exhibiting drug-resistance warrants the need to develop better vaccines or strategies for the prevention and treatment of TB [2]. The only available vaccine for TB is an attenuated form of *Mycobacterium bovis* named as *Bacillus Calmette–Guérin* (BCG) [3]. The efficacy of BCG is poor in populations with a high TB*-*burden [4]. BCG has proven its efficacy against childhood, but not adulthood manifestation of the disease, depicting an inability to generate enduring memory T cells against *Mtb*. BCG has lost the RD1 region from its genome. Although RD1 provides virulence in *Mtb,* it also evokes strong protective immunity against the bacterium signifying that BCG requires supplementation with certain *Mtb* proteins to improve its protective efficacy [5, 6]. In this regard, several prime-boost studies were conducted with BCG, such as protein and peptide-based subunit vaccines, live attenuated vaccines, and viral vectors with promising results [7].

Recently, we developed a lipidated promiscuous peptide vaccine comprising of the immunodominant CD4 and CD8 T cell epitopes of Acr1 and TB10.4 proteins of *Mtb* conjugated to TLR-2 ligand Pam2Cys [8, 9]. These constructs elicited enduring memory T cells response and showed better protection than BCG in mouse and Guinea pig TB models. Several advantages are associated with peptide vaccines, such as the selection of immunodominant moieties and the elimination of suppressive and auto-reactive portions of the antigen. However, there are certain issues associated with peptide vaccines due to its cost-effectiveness and synthesis for mass immunization. Hence, expressing the immunodominant epitopes inside the host could be an effective mode to eliminate the issues. An effective mode of expressing the epitopes would be the DNA vaccine strategy. A major advantage of DNA vaccines is that they are simpler to produce and store compared to conventional vaccines, making them less expensive. DNA vaccines can elicit the generation of both CD4 Th1 cells, CD8 T cells, and long-lasting immunity; the immune response that plays a cardinal role in protection against *Mtb* [10].

This encouraged us to design a DNA vaccine comprising of six CD4 T cells and CD8 T cells epitopes of latency, active and chronic stages of *Mtb*. To check the efficacy of the vaccine, it is important to use an animal model of TB and mice are very useful as their adaptive immune response is similar to humans. Hence, we immunized mice with DNA vaccine and observed induction of protective immune response that significantly reduced the frequency of bacterium in the animals exposed to *Mtb.* Furthermore, the vaccine considerably improved the efficacy of BCG to protect against *Mtb*. This vaccine may have future implications in protecting individuals from TB.

## Results

### Selection of T cell epitopes and construction of their sequence

**Fig. 1:**
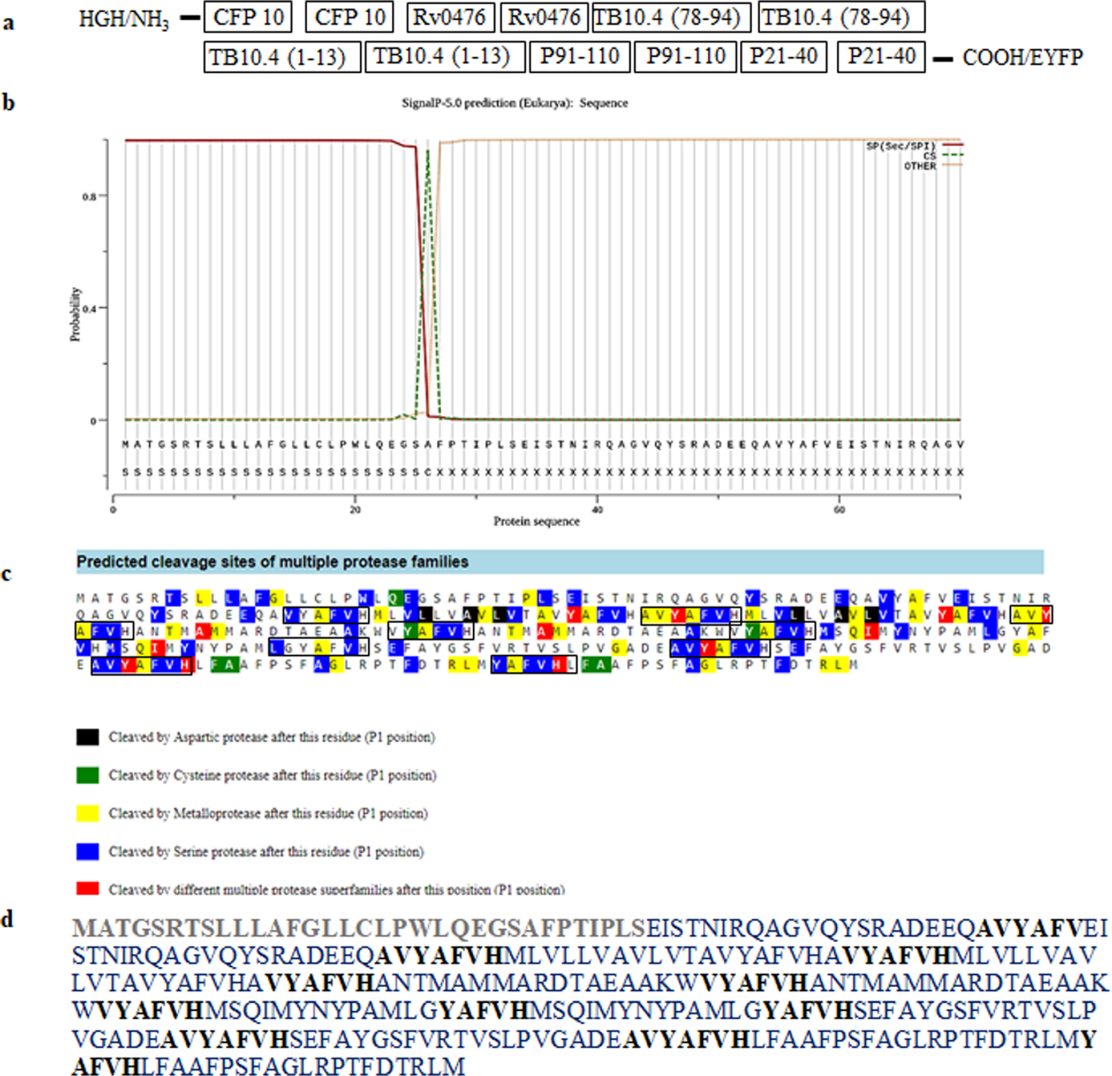
The Construction of C6 gene. **a** Diagrammatic representation of the arrangement of the C6 sequence. The addition of secretory property of C6 was analyzed by Signal-p 4.1. **b** The graph generated by Signal-p 4.1 shows secreting capacity into the host and cleavage site between positions 26 and 27. **c** PROSPER result of sequence analysis obtained after the addition of linker sequence (black box) between the epitopes exhibited higher sensitivity to proteases. **d** All six peptide sequences (blue) aligned in duplicates were attached by protease-sensitive linker sequence (Bold black) with N terminal secretory signal of human growth hormone (Bold gray)

To boost BCG efficacy, immunodominant T cell epitopes from different spectrums of TB as from latent, active, and chronic were selected from published literature [11–14]. The epitopes were promiscuous and showed the potential to elicit CD4 T cell and CD8 T cell response against *Mtb*. All the epitopes exhibited the ability to bind diverse HLA molecules. The six most immunodominant T cell epitopes were selected from Acr1, TB10.4, CFP10, and Rv0476 *Mtb* antigens (Table 1). The sequences were arranged in duplicates to increase the dose of the antigen (Fig. 1a). To segregate peptides during the process of antigen presentation, the chosen peptides were designed to have linkers that could be cleaved specifically by proteases present in antigen-presenting cells (APCs). To achieve this, the peptide sequences were checked for their sensitivity to proteases through in silico software PROSPER [15]. The Rv0476 peptide was found to be most sensitive to enzymatic cleavage and therefore was used as a linker between the epitopes (Supplementary Fig. 1a). The amino acid sequence AVYAFVH of epitope Rv0476_(1–19)_ was used as a linker between the epitopes. The initial two amino acid sequence is variable due to the presence of similarly charged amino acid sequence at the end of epitopes. To introduce a secretory signal in the protein, we added an N Terminal sequence of Human growth hormone (HGH), as a secretory signal [16]. The whole sequence (named and hereon referred to as C6) was further tested for its secretory capability in mammalian host cells. To analyze its release, Signal 4.1 server was used [17]. The Signal 4.1 server showed the N terminal secretory signal with secretion capability of protein and its cleavage site (Fig. 1b). The complete amino acid sequence was analyzed again in PROSPER to check the protease sensitivity of the linkers. The result indicated a higher sensitivity of linkers compared to the rest of the sequence (Fig. 1c). The final amino acid sequence of C6 was used for gene synthesis (Fig. 1d, Supplementary Fig. 1b).

**Table 1:** Selected T cell epitopes

Sr. No.	Protein	Sequence	TB spectrum	Reference
1	TB10.4_(1–13)_	MSQIMYNYPAMLG	Active	[12]
2	TB10.4_(78–94)_	ANTMAMMARDTAEAAKW	Active	[12]
3	Rv0476_(1–19)_	MLVLLVAVLVTAVYAFVHA	Active and latent	[13]
4	CFP10_(71–90)_	EISTNIRQAGVQYSRADEEQ	Active	[14]
5	Acr1_(91–110)_	SEFAYGSFVRTVSLPVGADE	Latent	[11]
6	Acr1_(21–40)_	LFAAFPSFAGLRPTFDTRLM	Latent	[11]

### Expression analysis of the selected T cell epitope-based gene construct

**Fig. 2:**
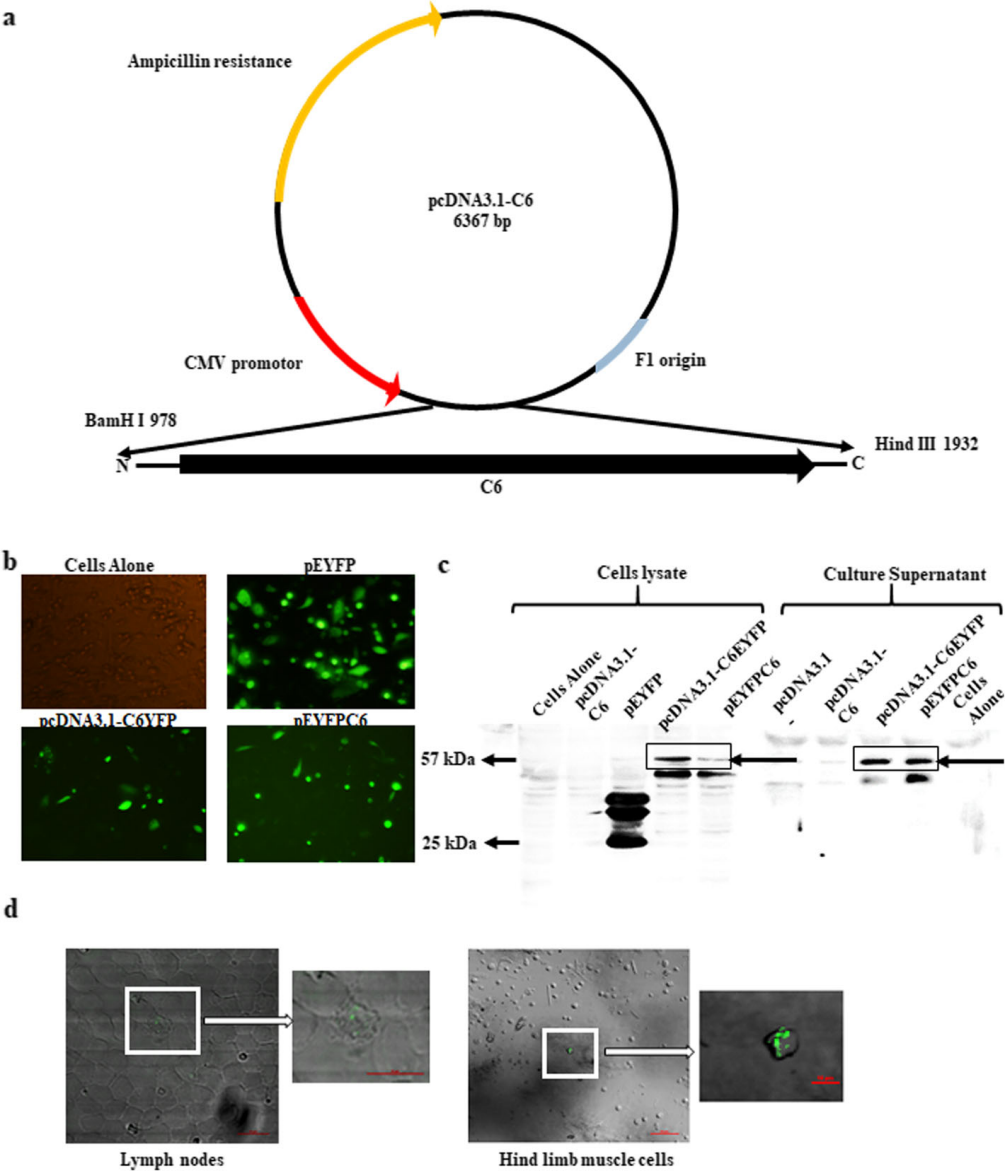
The constructed C6 gene expresses chimeric protein. **a** Vector map of DNA vaccine where C6 is cloned in between the BamHI and HindIII sites. **b** CHO cells were transfected with pcDNA3.1-C6, control pEYFP, and pEYFPC6 plasmids. After 24 h, the cells were analyzed under a fluorescent microscope. The transfected CHO cells with plasmids were harvested after 24 h of transfection. **c** The C6 expressing YFP was assessed in both cell lysate and SNs of transfected CHO cells through Western blotting. The protein bands in the inset signify a shift of the YFP band from 25 to 57 kDa. **d** C6 was inoculated into the hind limb of mice. Three days later, the cells from inguinal LNs and hind limb muscles were harvested and monitored by confocal microscopy for YFP expression. Lymph node cells and the hind limb cells showed YFP expression. The data are representative of 3 independent experiments

To use the C6 gene as a vaccine, a suitable vector must be used for its expression. Consequently, the C6 gene was cloned in pcDNA3.1(−) for immunization. Yellow fluorescent-tagged variants were generated for the expression analysis (Fig. 2a, Supplementary Fig. 2a-c). We transfected C6 gene in CHO cells and subsequently checked for its expression. The YFP-tagged C6 cells were observed under a fluorescence microscope (Fig. 2b). The transfected cells were further analyzed by flow cytometry. The decreased fluorescence intensity indicated the expression of the additional protein (C6) with YFP (Supplementary Fig. 3a, b).

Further, total cell lysate of transfected CHO cells and culture SNs precipitate was used for Western blotting. The cells expressing YFP with C6 were observed as a band of 57 kDa molecular weight (mwt) in the blot. The YFP alone appeared at mwt of 25 kDa. The expression of YFP attached with C6 indicates the secretion of protein in SN (Fig. 2c). Both fluorescent microscopy and Western blotting results confirmed in vitro expression of C6.

It is important for a DNA vaccine to get expressed in host cells and subsequently produce antigens and prime the cells of the immune system. To accomplish this, mice were immunized intramuscular (i.m.) with plasmid C6YFP and control vector. The animals were rested for 3d and YFP expression was checked in the lymph nodes and hind limb muscles via confocal microscopy. We observed YFP expression in both cell types, thus confirming the expression of C6 in vivo (Fig. 2d).

### Immunization with BCG + C6 augments the T cell memory

**Fig. 3:**
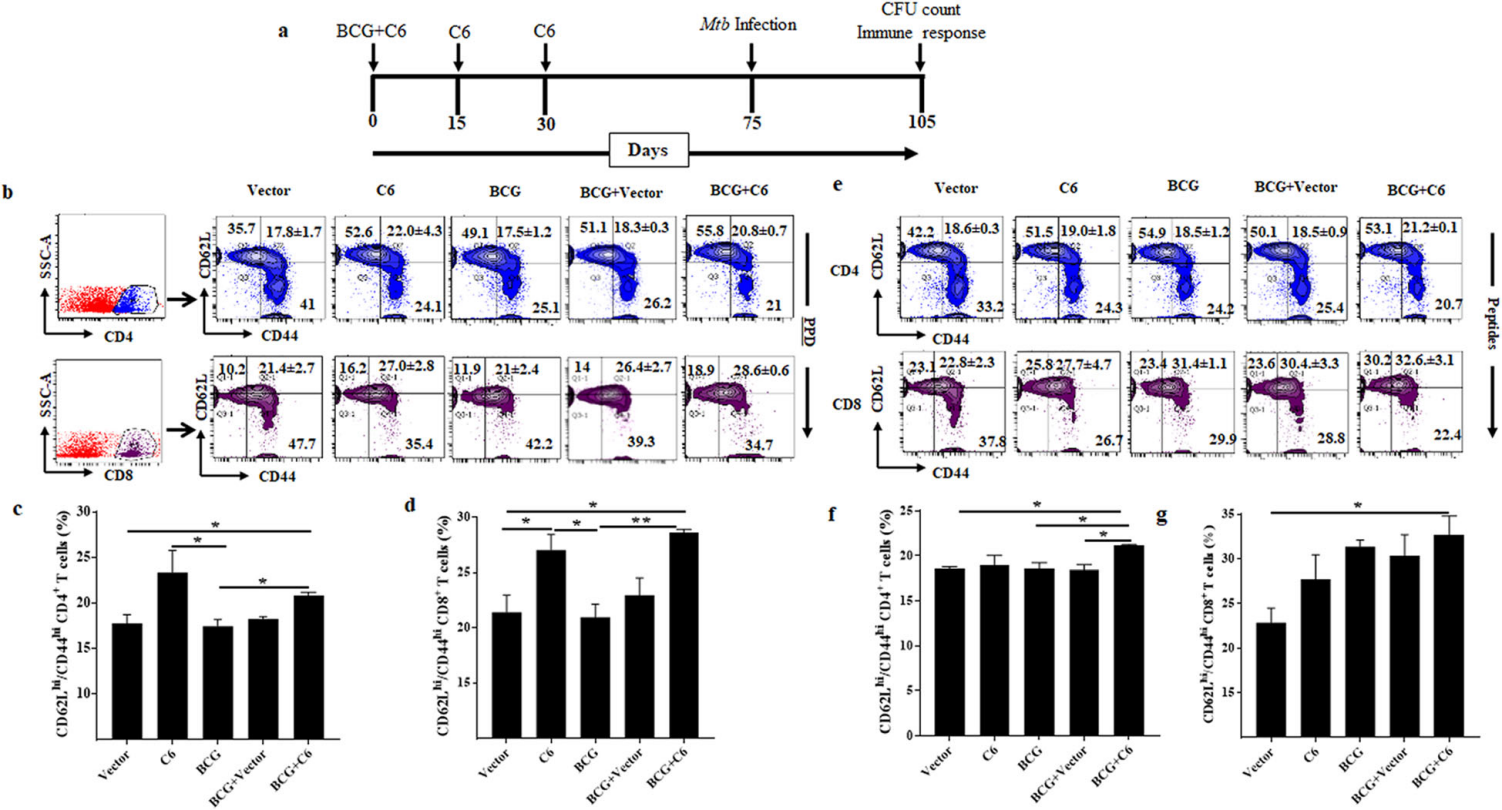
Immunization with BCG and C6 augments memory CD4 T cell and CD8 T cell response. **a** Mice injected with C6 and control groups with BCG, C6 + BCG, BCG + Vector, plasmid, and challenged with *Mtb*. Thirty days later, lymphocytes of spleen and lymph nodes were harvested and in vitro stimulated with PPD (25 μg/ml) and C6 peptides. **b, e** The contour plots depict the percent population of CD62L^hi^CD44^hi^ central memory CD4 T cells and CD8 T cells upon (**a**) PPD and (**b**) C6 peptides stimulation. Bar diagrams are representative of percent population of CD4 and CD8 T cell upon (**c**, **d**) PPD and (**f**, **g**) peptide stimulation. The number in the inset signifies the percentage of cells. Data are the representative of three independent experiments. * *p* ≤ 0.05, ** *p* ≤ 0.005

After confirmation of YFP expression, we wanted to check the viability of C6 as a vaccine candidate and its ability to enhance the efficacy of BCG. Therefore, we immunized mice with BCG, C6, and controls (Negative, vector control), subsequently infecting them with Mtb aerosol. After 30d we assessed each group’s immune response (Fig. 3a). Generation of persistent memory T cells against a pathogen is essential for any successful vaccine. PPD is a rich source of *Mtb* antigens and is used to check the immune response against *Mtb*. We have used 6 different T cells epitopes of *Mtb* in DNA vaccine to induce an immune response against these epitopes. So, it is important to check the immune response against these epitopes as well. Therefore, we examined T cell memory response following C6 immunization. Spleen and lymph node-derived lymphocytes were stimulated in vitro either with PPD or mixture of peptides to monitor the expression of memory markers CD44^hi^ and CD62L^hi^ on CD4 and CD8 T cells (Fig. 3b-g). We observed an increased percentage of CD62L^hi^CD44^hi^ expressing memory CD4 T cells (PPD: *p* < 0.05) and CD8 T cells (PPD: *p* < 0.05) in C6 immunized mice, as compared to BCG on in vitro stimulation with PPD (Fig. 3b-d). However, in vitro stimulation with C6 peptides showed a non-significant increase in memory CD4 and CD8 T cell frequency (Fig. 3e-g). Furthermore, we observed that C6 bolstered the generation of memory CD4 T cells (PPD: *p* < 0.05, Peptides: (*p* < 0.005) and CD8 T cells (PPD: *p* < 0.05, Peptides: (*p* < 0.05) in the group that was vaccinated with BCG (BCG + C6) compared to BCG alone. Furthermore, we observed an expansion in the pool of memory T cells in the lungs of the same animals (Supplementary Fig. 4a-d). These results indicate the potential of C6 to not only expand memory CD4 T cells and CD8 T cells but to also boost memory T cell generation associated with BCG.

### Combinatorial administration of BCG + C6 improves the secretion of IFN-γ, TNF-α, and inhibits IL-10 release

**Fig. 4:**
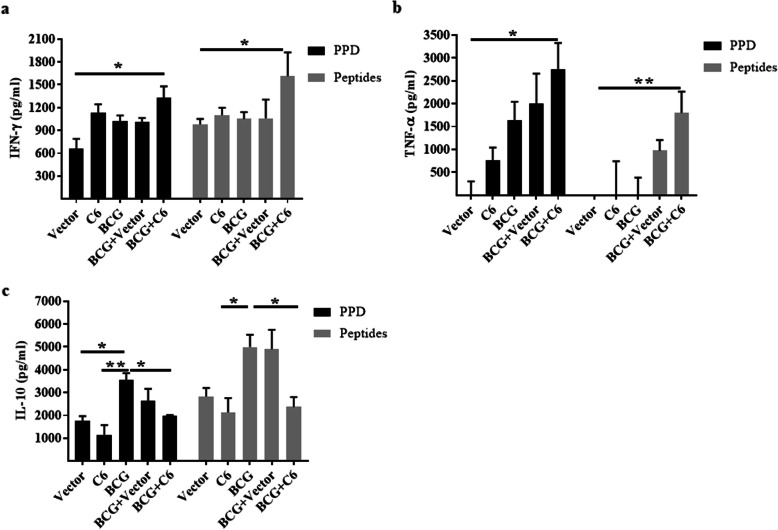
Immunization with C6 promotes IFN-γ and TNF-α secretion and decreases IL-10 release. Thirty days later of the *Mtb* challenge, lymphocytes of spleen and lymph nodes isolated from the BCG + C6 immunized and control animals were in vitro stimulated for 72 h with PPD and peptides. Later, culture SNs were harvested and monitored by ELISA for the production of **a** IFN-γ; **b** TNF-α; and **c** IL-10. The data shown as mean ± SEM are from triplicate wells of two independent experiments. * *p* < 0.05, ** *p* < 0.005, *** *p* < 0.0005, **** *p* < 0.0001

Mounting of a Th1 immune response is crucial for combatting *Mtb* infection [18]. IFN-γ and TNF-α released by Th1 cells both serve an important function in *Mtb* infection by activating phagocytic cells [19]. Therefore, we checked if BCG + C6 immunization can enhance IFN-γ and TNF-α secretion by in vitro stimulation with PPD and peptides. It was found that immunization with BCG + C6 significantly increased the production of IFN-γ (PPD: *p* < 0.05, peptides: *p* < 0.05) and TNF-α (PPD: *p* < 0.05, peptides: *p* < 0.005) as compared to a vector control. We found that BCG vaccination alone showed no significant increase in such cytokine production (Fig. 4a, b). C6 alone showed insignificant increases in IFN-γ and TNF-α release. Surprisingly, we observed an increased level of IL-10 (*p* < 0.05) in BCG immunized group compared to the vector control. In contrast, IL-10 production in BCG + C6 immunized animals was significantly lower (*p* < 0.05) upon immunization in comparison to BCG control, indicating a capability to promote Th1 response (Fig. 4c). These results support the generation of Th1 immunity against *Mtb* upon BCG + C6 immunization.

### Immunization with C6 promotes the activation of antigen-presenting cells

**Fig. 5:**
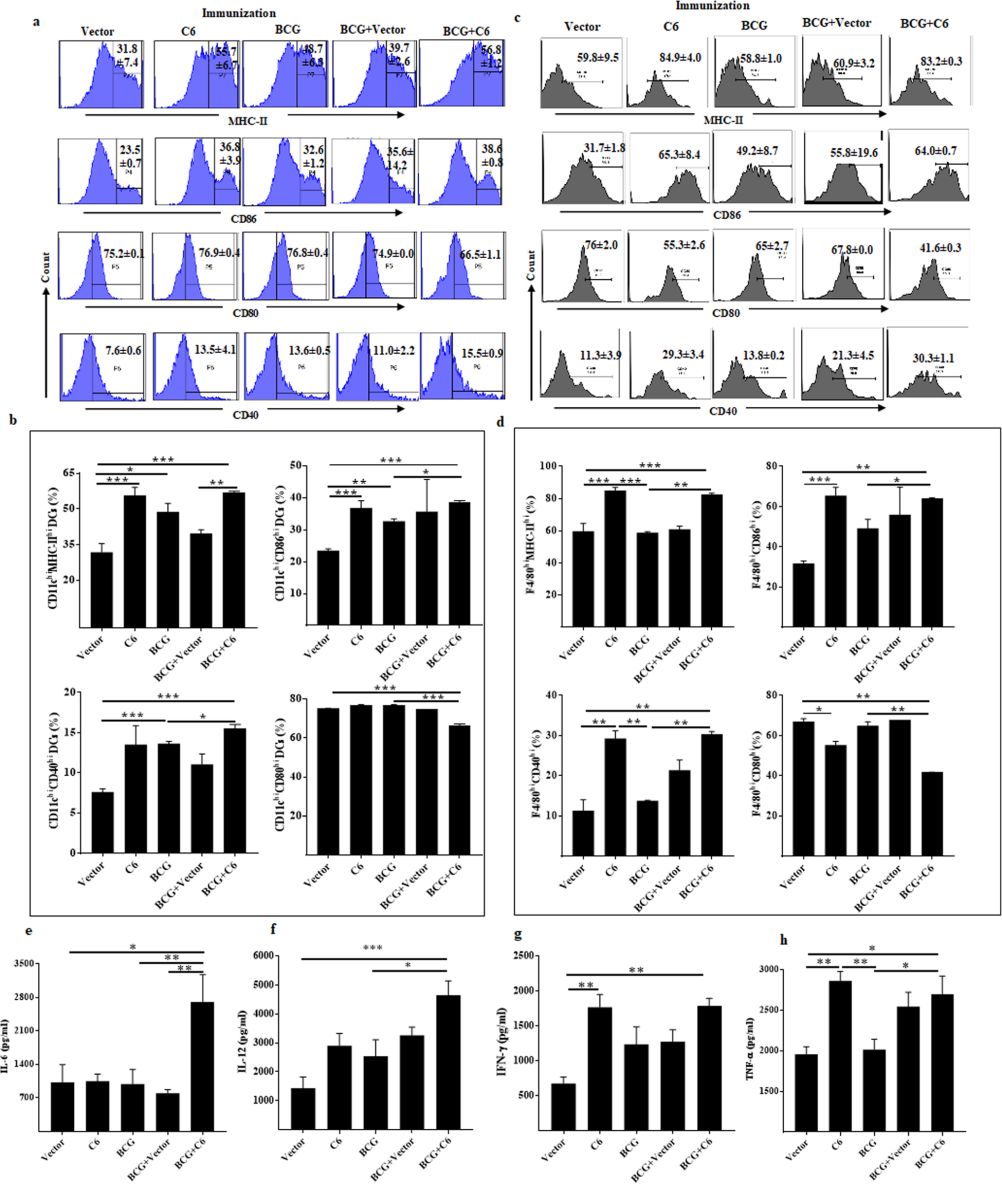
Animals immunized with C6 show better activation of DCs and macrophages and augment the secretion of IL-6 and IL-12. Cells isolated from the lungs of the C6 immunized mice and control groups (vector, BCG, BCG + Vector C6 + BCG) were in vitro stimulated with LPS (1 μg/ml) for 24 h. **a**, **c** histogram and their respective (**b, d**) bar diagram signify the percent population of MHC-II^hi^, CD80^hi^, CD86^hi^, CD40^hi^ expression on (**a**) DCs; (**c**) macrophages. Data (means±SEM) are represented as percent positive cells of two independent experiments. **e**-**f** The culture SNs were harvested from LPS stimulated lymphocyte cultures to estimate the secretion of **e** IL-6; **f** IL-12; **g** IFN-γ; **h** TNF-α. The data shown as mean ± SEM are obtained from triplicate wells of two independent experiments. * *p* ≤ 0.05, ** *p* ≤ 0.005, *** *p* ≤ 0.0005

The immune system responsible for the clearance of pathogens is primarily antigen-presenting cells (APCs), such as macrophages and dendritic cells (DCs) [20, 21]. *Mtb* is known to modulate APCs to prevent the activation and expression of MHC and co-stimulatory molecules [20–22]. Therefore, we wanted to examine the immune status of APCs in the lungs upon C6 administration from *Mtb* infected mice. It was found that upon stimulation with LPS, the percentage of MHC-II^hi^, CD86^hi^, and CD40^hi^ expressing DCs isolated from the lungs was greater in C6 and BCG + C6 inoculations as compared to their respective controls (vector, BCG) (Fig. 5a, b). Similarly, the percentage of MHC-II^hi^, CD86^hi^, and CD40^hi^ expressing macrophages were also higher in C6 and BCG + C6 groups than controls (Fig. 5c, d). However, there was a considerable decrease in the percent population of CD80^hi^ expressing DCs and macrophages. Similar results were observed with DCs and macrophages isolated from the spleen and lymph nodes (Supplementary Fig. 5a-e).

Among several cytokines produced by activated DCs, IL-6 and IL-12 are quite crucial since they play a fundamental role in the differentiation of naïve CD4 T cells into Th1 cells [20, 21] Interestingly, we observed that when compared to control groups (vector, BCG), mice inoculated with BCG + C6 exhibited significantly higher production of IL-6 (*p* < 0.05, *p* < 0.01) and IL-12 (*p* < 0.001) (Fig. 5e, f). Upon activation, dendritic cells and macrophages produce pro-inflammatory cytokines [23–26]. We monitored IFN-γ and TNF-α in LPS stimulated lymphocytes culture supernatant and observed elevated IFN-γ (*p* < 0.005) and TNF-α (*p* < 0.05) in the BCG + C6 group compared to vector. Mice treated with BCG did not elicit the same response (Fig. 5g, h). These results signify the important role of C6 in generating a favourable immune response for clearing *Mtb* infection.

### BCG administration with C6 significantly reduced *Mtb* burden and disease pathology

**Fig. 6:**
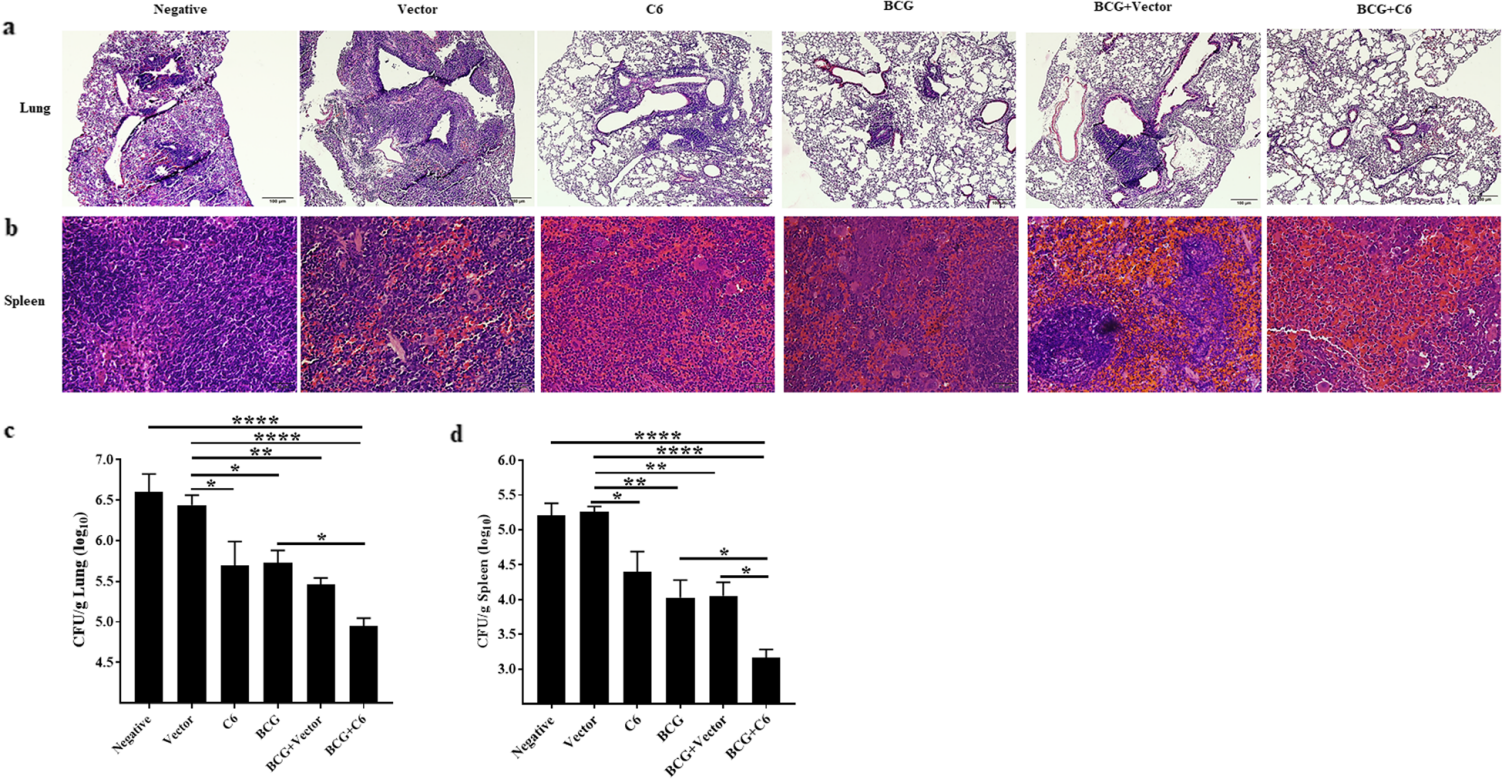
Prime-boosting with C6 + BCG protects against Mtb infection. BCG + C6 injected mice were boosted with C6 and control groups with vector alone or with PBS at an interval of 15d. After 45d, mice were infected with *Mtb*. Thirty days later, the *Mtb* burden in the lungs and spleen was enumerated by CFUs. Histopathology of the **a** lung (10x) and **b** spleen (40x) sections stained with hematoxylin-eosin. **c**, **d** Bar diagram signifies the CFUs per gram of lung and spleen tissues. The intensity of the blue colour indicates infiltration and consolidation. Data are representative of 3 independent experiments with 3 animals in each group. The results displayed as bar diagram (mean ± SEM) are pooled data from 3 independent experiments. * *p* ≤ 0.05, ** *p* ≤ 0.005, **** *p* ≤ 0.0001

We further assessed the protective role of C6 in reducing the pleural mycobacterium burden in *Mtb*-infected animals. C6 injected mice were boosted twice with C6. After 45d, they were aerosol challenged with *Mtb*. *Mtb* burden in the lungs and spleen was assessed 30d after infection. Histopathology analysis revealed a reduction in disease pathology in both the lungs and spleen of C6 and BCG + C6 groups, as compared to negative and vector groups. There was lower peribranchial lymphocyte infiltration in the lungs as well as a decreased number of follicles in the spleen of the C6 and BCG + C6 vaccinated mice (Fig. 6a, b). Furthermore, C6 vaccinated animals showed a significant reduction (*p* < 0.05) in *Mtb* CFUs, as compared to control unvaccinated negative and vector inoculated groups (Fig. 6c). Restriction in the dissemination of *Mtb* to the spleen was observed and confirmed by a significant reduction in *Mtb* CFU (Fig. 6d). It was surprising to note that although C6 induced a better immune response (Figs. 2, 3, 4 and 5) than BCG vaccinated mice, the decrease in CFUs was similar to BCG vaccinated mice. Furthermore, BCG + C6 animals exhibited a significantly better decline in the bacterial burden (*p* < 0.05), when compared with the BCG vaccinated mice, indicating a synergistic effect between both the vaccines. These results indicate that the efficiency of BCG protection can be considerably bolstered by co-administration of the C6 construct expressing the immunodominant T cell epitopes of *Mtb*.

## Discussion

The poor performance of BCG in TB-endemic areas can be rationalized with multiple explanations. BCG protects the childhood but not adult manifestation of TB [27–30]. Signifying that it lacks the antigenic repertoire that is required in inducing long-lasting protective memory T cells. Consequently, supplementing *Mtb* antigenic epitopes in BCG may bolster its performance. Therefore, in the current study, we selected multiple epitopes from latent, active, and chronic stages of TB and synthesized a DNA vaccine to check its efficiency either alone or in a combination of BCG.

It has been previously reported that the Acr1 protein provides enhanced protective efficacy when overexpressed in BCG [31]. However, Acr1 impairs the maturation and functionality of DCs and supports the intracellular survival of *Mtb* [22, 32]. Similarly, Rv2626c protein has been shown to protect against *Mtb*, as well as modulate the functionality of macrophages and assist in the escape of pathogen [33, 34]. Hence, the expression of CD4 T cell and CD8 T cell epitopes in a DNA vaccine with BCG may be a better approach to combat TB. Promiscuous T cell epitopes have enough potential to bind diverse HLA alleles and evoke T cell activation without requiring extensive antigen processing by APCs [8, 35]. However, peptide vaccines are weak immunogens and thus require adjuvants to elicit optimum activation of T cells.

Therefore, we selected and expressed multiple T cell epitopes from the latent, active, and chronic stages of TB in the DNA vector to overcome the limits associated with BCG. DNA vaccines can induce both CD4 T cells and CD8 T cell responses against the expressing antigens [10]. This ability has led to the development of many veterinary vaccines and human clinical trials involving Zika, HIV, dengue, and cancer diseases [36–40]. In connection with TB, DNA vaccines have shown the potential to combat infection [41–46]. In this light, we selected six promiscuous CD4 T cell and CD8 T cell epitopes from the different *Mtb* proteins [11–14] and cloned them into pcDNA3.1(−) plasmid. The novelty of the C6 construct is that it has 2 copies of each epitope linked with protease-sensitive amino acid sequences. This allows for APC-mediated protease cleavage and eventual release from C6 expressing cells with the help of a secretory signal (Fig. 1). Furthermore, the use of a plasmid vector helps in the elicitation of the CD8 T cell response [47]. We generated the YFP reporter construct with C6 by cloning it into the plasmid pcDNA3.1(−) and pEYFP to evaluate its expression and secretion. We confirmed the expression of C6 along with YFP in vitro into the CHO cells by fluorescence imaging, as well as Western blotting (Fig. 2). Also, it was important for the construct to be expressed in vivo to evoke an immune response. We immunized animals with C6 and observed YFP expressing cells in the mice, thus confirming the expression of C6. Furthermore, we examined if the C6 vaccine could enhance the efficacy of BCG. We evaluated the boosting capacity of C6 in BCG vaccinated mice. We noticed following major outcomes on C6 vaccination: (1) generation of memory CD4 T cell and CD8 T cells; (2) enhancement in Th1 responses, as evidenced by the predominant secretion of IFN-γ and TNF-α; (3) promotion of the activation of APCs; (4) boosting of protective efficacy of BCG against *Mtb*.

Immunological memory is an indispensable feature of adaptive immunity that protects organisms from subsequent infections [48]. Moreover, it is a fundamental feature of a successful vaccine [49]. The generation of short-term memory T cells is one of the reasons for the failure of BCG to impart protection against *Mtb* in the vaccinated adult population [50]. Remarkably, BCG generates better memory CD4 T cells and CD8 T cells response with the addition of memory response by C6 (Fig. 3). The enhancement in memory response could be observed due to the generation epitope-specific immune response. It has been reported that resting T cell population with naïve phenotype i.e. CD62L^hi^/CD44^lo^ can confer protection against *Mtb* [51, 52]. The increase in the resting population upon C6 administration denotes the enhancement in the generation of resting population, which is important for recall response. Intriguingly, C6 potentiated the capacity BCG in augmenting CD62L^hi^/CD44^lo^. Decreased the percentage of CD44^hi^/CD62L^lo^ cells in C6 and BCG + C6 indicates the capability of effector cells to transit towards memory cell which is poorly associated with BCG and explain its failure in persistent *Mtb* infections [53–55].

CD4 T cell subsets express distinct cytokines and transcription factors, thereby responding to different pathogens. Th1 cells protects against *Mtb* by secreting IFN-γ and TNF-α and stimulating macrophages to kill intracellular pathogens [56, 57]. The importance of IFN-γ is illustrated as its absence enhances *Mtb* susceptibility, mortality, and defects in macrophage activation [58]. For initiation and maintenance of defence against *Mtb*, TNF-α plays a crucial role in reactivation of latent tuberculosis of rheumatoid arthritis patients during the neutralization by the anti-TNF antibody [59]. Therefore, the generation of Th1 immunity by a vaccine is quite crucial to protect against TB. The elicitation of higher yield of IFN-γ and TNF-α by BCG + C6 denotes its potential to generate Th1 response (Fig. 4). IL-10 is produced by Th2 cells and can reciprocally regulate the generation of Th1 cells as well as the macrophage and DCs to activate Th1 cells [60, 61]. Furthermore, it has been shown that BCG infected dendritic cells generate IL-10 producing T cells [62]. we observed elevated expression of IL-10 in the BCG immunized group. In contrast, C6 alone and along with BCG immunization declined the IL-10 secretion, indicating its ability to promote Th1 cells.

The importance of antigen-presenting cells (APCs), such as DCs and macrophages, in the protection against TB is well elucidated [21]. Besides phagocytosing and killing the pathogens, these cells simultaneously process and present the pathogenic components to activate and differentiate T cells into effector and memory T cells. These activated T cells help to bolster the function of APCs to release cytokines like IFN-γ and TNF-α [49, 56, 57].

*Mtb* can modulate APCs to restrict the generation of adaptive immunity. *Mtb* inhibits the maturation of APCs by preventing antigen presentation through MHC along with costimulatory molecules such as CD86, CD40, and CD80 masks the ability to activate antigen-specific T cells [63, 64]. Interestingly, DNA vaccines can activate APCs by interacting with TLR9 [65]. It is noteworthy to mention here that the activation of DCs and macrophages in BCG + C6 immunized animals were higher, as evidenced by the increased percentage of MHC-II^hi^, CD86^hi,^ and CD40^hi^ costimulatory molecules expressing cells (Fig. 5). CD80 preferably interacts with CTLA-4 molecule of T cells and weakly with CD28 and is linked with the generation of anergy and tolerogenic T cells [66]. The reduction of CD80^hi^ percent population of DCs and macrophage in BCG + C6 supports the generation of pathogenic T cells rather than tolerance. To activate T cells and generate effector and memory T cells, DCs and macrophages produce IL-6 and IL-12. Thus, activation and secretion of IL-6 and IL-12 are crucial for the APCs [18]. Increased production of IL-6 and IL-12 in the BCG + C6 group indicates the enhanced capability of DCs and macrophages to activate T cells. Similarly, the role of IFN-γ and TNF-α has been correlated with the functionality of DCs and macrophages [67, 68]. Therefore, the production of IFN-γ and TNF-α indicates the activation of DCs and macrophage.

Apart from the generation of optimum activation of the immune system, a cardinal feature of a vaccine is to restrict infection. During the progression of TB, the infiltration of inflammatory mononuclear cells leads to the development of granulomas; a habitable niche for *Mtb* [69]. Furthermore, acute bronchopneumonia and necrotizing granulomas have been correlated with the pathology of pulmonary TB [70]. Remarkably, we observed a decrease in the *Mtb* burden and disease pathology of the lungs of the C6 administered group and augmented the potency of BCG (Fig. 6). C6 prevented the dissemination of *Mtb,* as depicted by a decrease in the bacterial burden in the spleen. The reduced bacterial burden in the vaccinated group indicates the protective efficacy of C6 with BCG.

The C6 vaccination along with BCG has improved the protection against the Mtb in the mice model of TB. Its protective efficacy has been achieved by the generation of memory T cells against Mtb. These T cells can activate DCs and macrophages with the help of IFN- and TNF-. Moreover, the DCs and macrophages in immunized animals were not affected by the suppressive ability of *Mtb* and could produce IL-6 and IL-12 to further activate T cells. All these together declined the *Mtb* burden in the vaccinated group of animals compared to controls.

## Conclusion

TB has been ranked as one of the world’s most deadly diseases. Cumbersome therapeutic strategies, drug-resistant strains, and failures of the common BCG vaccine all further the necessity of efficacious vaccine development. Subunit vaccines have provided a benefit over whole cell-based vaccines [8, 9, 71]. Overall, our studies indicate that a multi T cell epitope-based DNA vaccine substantially enhances the immunity and protection of BCG against *Mtb*. These results affirm the potential viability of C6 as a vaccine candidate in the effort to control TB.

## Methods

### Mice

BALB/c and C57BL/6 female mice (6–8 weeks, 16–18 g) were obtained from the Animal House Facility, CSIR-Institute of Microbial Technology, Chandigarh (IMTECH) and kept in Biosafety level 3 laboratory in CSIR-Institute of Microbial Technology, Chandigarh (IMTECH) for experimental procedures.

### Bacteria and cell lines

The *Escherichia coli (E. coli)* DH5α strain was grown in LB media and used in this study for cloning and purification of plasmids. BCG Danish strain (Serum Institute of India PVT. LTD., India) used for immunization. *Mtb* H37Rv strain was grown in 7H9 + 10%OADC and preserved as 10% glycerol stock at − 80 °C to be used for infection respectively. CHO cell line was used for the transfection studies.

### Reagents

All the reagents and primers were purchased from Sigma (St. Louis, MO) and antibodies from eBiosciences (San Diego, CA), Restriction, and ligase enzymes were from New England Biolabs (Ipswich, MA), further unless and otherwise mentioned. Bacterial media were purchased from Himedia (Mumbai, India).

### T cell epitopes selection, cloning, and expression

The promiscuous T cell epitope selection was based on binding to multiple HLA alleles. We selected 6 promiscuous CD4 T cells and CD8 T cell epitope peptides from the literature. The peptide sequences were arranged in duplicates and linked with a protease-sensitive amino acid sequence (AVYAFVH). An N-terminal human growth hormone (HGH) secretory signal was linked for the secretion of the protein from the host cells. The chimera gene (C6) for the protein was synthesized (GenScript, Piscataway, NJ). To use the C6 gene as a vaccine, a suitable vector must be used for the expression. Consequently, to utilize as a DNA vaccine, vector pcDNA3.1- was used. The synthesized gene was cloned into the pcDNA3.1- vector at the site of BamHI and HindIII and transformed into *E. coli* DH5α for multiplication and purification of the plasmid. The presence of the C6 gene in the plasmid was confirmed through colony PCR and agarose gel electrophoresis.

Later, C6 was cloned into the pEYFP-N1 vector at the site of the NheI and HindIII site to generate a YFP tagged protein for the expression confirmation of gene in the host cells. pEYFP-C6 was transformed into *E. coli* and kanamycin-resistant colonies were screened through colony PCR and agarose gel electrophoresis. To further confirm the expression of C6 in pcDNA3.1- vector, the C6YFP gene was amplified and cloned into the NheI and NotI site of pcDNA3.1- and transformed into *E. coli* and positive colonies were selected through PCR and agarose gel electrophoresis. All the plasmids for the use of immunization and transfections were isolated through the Triton X-114 method [72].

The CHO cell line was transfected with plasmids by using lipofectamine 2000 (Invitrogen, Carlsbad, CA). The standard manufacturer protocol was followed for the transfection. Transfected cells were used for direct observation under a fluorescent microscope, western blotting, and FACS analysis.

### Western blotting

Transfected CHO cell lysate was prepared by harvesting, washing, and lysis in lysis buffer (RIPA buffer, protease, and phosphatase inhibitor cocktail). The culture supernatants (SN) were precipitated through Acetone precipitation. Briefly, five times a volume of 80% chilled acetone was added to the SN and incubated overnight at − 20 °C. Later, SN was pelleted at 10000 g for 15 min at 4 °C. The pellets were washed twice with 80% chilled acetone and air-dried for 45 min at RT. Pellets were dissolved into PBS. The SNs of the cell lysate and culture SNs were estimated and equal concentration was subjected to SDS-PAGE. After transfer onto nitrocellulose membrane and blocking, the membranes were immunoblotted with Abs against YFP. Blots were developed using a chemiluminescence kit (Thermo Scientific, Waltham, MA). Chemiluminescence was detected by ImageQuant LAS 4000 (GE life sciences, UK).

### Animal immunization

To study the in vivo expression of C6, C57BL/6 mice were vaccinated with 100 μg of C6YFP, and inguinal LNs and hind limbs were isolated 3d later to check YFP expressing cells. For the immunological studies, BALB/c mice were immunized subcutaneously (s.c.) at the base of the tail with BCG (10^6^ CFU/animal) along with intramuscularly (i.m.) in the hind limb with 100 μg/animal of C6 and controls (pcDNA3.1-, C6, BCG and negative) in PBS as 3 mice in a group. Two booster doses of DNA vaccine were given at the interval of 2 weeks. Later, mice were euthanized for organ analysis.

### Aerosol infection and bacterial burden in the lungs and spleen

Immunized mice were rested for 30d and aerosol challenged with 100 CFU of live *Mtb* by Inhalation Exposure System (GlasCol, LLC, Terre Haute, IN). Thirty days after the infection, animals were sacrificed and bacterial burden in lungs and spleen were determined by inoculation of tissue homogenates on 7H11 plates. Lungs and spleen sections were also preserved in 1% formalin in PBS for the histopathological analysis by hematoxylin and eosin staining.

### Spleen and lung lymphocyte culture

Spleen, lymph nodes (LNs), and lung cells were prepared by crushing of tissues followed by RBC lysis. Lymphocytes (2 × 10^5^/well) isolated from spleens/LNs or lungs were cultured in 96-well U bottom plates and stimulated with PPD (25 μg/ml) and 5 C6 peptides (5 μg/ml each) as Rv0476_(1–19)_ was unable to synthesize. For DCs and macrophages activation status studies, cells were stimulated with LPS (1 μg/ml) for 24 h.

### Flowcytometry

For phenotypic analysis of T cells, the PPD and peptides stimulated lungs and spleen/LNs cells were analysed by flow cytometry. Lymphocytes culture were harvested and stained with fluorochrome tagged anti-CD4-PE, CD8-APCCy7, CD62L-FITC, CD44-PerCPCy5.5, CD11c-PECy7, F4/80-APC, CD86-PE, CD80-FITC, CD40-PECy5, and MHC-II-PerCPCy5.5abs (BD Biosciences, San Jose, CA). Briefly, lymphocytes were harvested in tubes and washed with FACS buffer (PBS + 2%FCS). Cells were Fc blocked using anti-mouse CD16/CD32 Ab. Later, stained with fluorochrome-labelled Abs. After staining, cells were fixed by using 1% paraformaldehyde in FACS buffer. Cells were acquired in BD-FACS Aria III and BD-FACS Accuri (BD, Franklin Lakes, NJ). The analysis was performed using BD-FACS DIVA, BD-C6, and Flowjo software (BD, Franklin Lakes, NJ).

### Cytokine ELISA

The expression of different cytokines in the culture supernatants from PPD and peptide stimulated lymphocyte cultures were monitored by sandwich ELISA. Briefly, primary anti-cytokine antibodies were coated on 96-well plates at 4 °C overnight. Next, wells were blocked with 2% BSA solution for 2 h and incubated overnight at 4 °C with the culture supernatants. Later, plates were incubated 2 h with biotinylated secondary antibodies and 45 min with streptavidin-HRP conjugates. OPD-H_2_O_2_ substrates were used to determine the concentration of cytokines along with standards by obtaining reading at 595 nm.

### Bioinformatic tools

For the bioinformatic analysis Ligation calculator (http://www.insilico.uni-duesseldorf.de/Lig_Input.html), PROSPER (https://prosper.erc.monash.edu.au/), SignalP 4.1 Server (http://www.cbs.dtu.dk/services/SignalP/) and PlasMapper (http://wishart.biology.ualberta.ca/PlasMapper/) tools were used.

### Statistics

All the statistical analysis was performed as One-way ANOVA with Tukey’s test in Graph Pad Prism (GraphPad Software, La Jolla, CA).

## Supplementary information

**Additional file 1: Supplementary Figure 1**. *C6 gene construction*. (a) All the peptide sequences were analyzed by PROSPER software to check their sensitivity to proteases. Different colors indicate different proteases. ‘Red colour’ designates the sequence of Rv0476, which was found to be most sensitive to protease degradation. (b) The complete gene sequence of C6 (963bp) is shown that was used for the synthesis of the DNA vaccine. **Supplementary Figure 2**. *C6 was cloned successfully into the plasmid*. (a) The C6 gene was cloned into pcDNA3.1- and transformed into *E. coli*. The agarose gel electrophoresis confirms the presence of the C6 gene with the additional size of the restriction site and extra-base pairs used for restriction digestion. C6: positive control, lane 1–8: different *E. coli* clones. The vector map of pcDNA3.1-C6 with the site was used for cloning. (b) The C6 gene was cloned into a pEYFP-N1 plasmid and transformed into *E. coli*. The agarose gel electrophoresis confirms positive *E. coli* colonies with the C6 gene. Lane 1–10 indicates the different *E. coli* colonies and C indicates the positive control of the C6 gene. The vector map of pEYFP-C6 was used for cloning and as an open reading frame. (c) The C6YFP gene was cloned into pcDNA3.1- plasmid and transformed into *E. coli*. The agarose gel electrophoresis confirms positive *E. coli* colonies with the C6 gene. Lane 1–6 indicate the different *E. coli* colonies. pEYFP-C6 vector was used as a positive control. The vector map of pcDNA3.1-C6YFP with the site was used for cloning and as an open reading frame. **Supplementary Figure 3**. *C6-YFP expression in CHO cells*. (a) The C6-YFP transfected CHO cells were analyzed by flow cytometry for their fluorescence intensity (YFP) and represented as histogram plots. Data in the inset represent the mean fluorescence intensity (MFI). (b) The bar diagram represents the integrated mean fluorescence intensity (iMFI) of the transfected cells, further, confirm the presence of C6. Data expressed as mean ± SEM are representative of 2 independent experiments. * *p* ≤ 0.05. **Supplementary Figure 4**. *Co-immunization of BCG with C6 augments memory T cell response*. Lymphocytes from the lungs were isolated from the BCG + C6 injected and control animals. Lymphocytes were in vitro stimulated with PPD and peptides. (a, c) Contour plots show the percent population of CD62L^hi^CD44^hi^ central memory CD4 T cells and CD8 T cells, stimulated with (a) PPD and (c) peptides and represented as (b, d) bar diagrams. Data are from two independent experiments and represented as mean ± SEM. **Supplementary Figure 5**. *Prime boosting with BCG and C6 enhances the activation of DCs and macrophages*. Lymphocytes were isolated from spleen and LNs from the BCG + C6 immunized and control animals. Cells were in vitro stimulated with LPS (1 μg/ml) for 24 h. (a) Gating strategy used for DCs and macrophage cells. (b, c) histogram and their respective (d, e) bar diagram signify the percent population of CD86^hi^, CD40^hi^, MHC-II^hi^, and CD80^hi^ expressing (b) DCs and (c) macrophages. Data (means±SEM) represented as percent positive cells are of two independent experiments. *p ≤ 0.05, **p ≤ 0.005, ***p ≤ 0.0005, ****p ≤ 0.0001.

## Data Availability

All data generated or analysed during this study are included in this article (and its supplementary information files). All the datasets used and analysed are available upon reasonable request from the corresponding author.

## Abbreviation

C6: pcDNA3.1- with multiple T cell epitope of *Mtb*
