# Supplementary material for: A multiple T cell epitope comprising DNA vaccine boosts the protective efficacy of Bacillus Calmette–Guérin (BCG) against Mycobacterium tuberculosis
Source: BMC Infect Dis. 2020 Sep 17;20:677. doi: 10.1186/s12879-020-05372-1 (PMC7495405; doi:10.1186/s12879-020-05372-1)
Supplement: Supplementary file 1 — Additional file 1: Supplementary Figure 1. C6 gene construction. (a) All the peptide sequences were analyzed by PROSPER software to check their sensitivity to proteases. Different colors indicate different proteases. ‘Red colour’ designates the sequence of Rv0476, which was found to be most sensitive to protease degradation. (b) The complete gene sequence of C6 (963bp) is shown that was used for the synthesis of the DNA vaccine. Supplementary Figure 2. C6 was cloned successfully into the plasmid. (a) The C6 gene was cloned into pcDNA3.1- and transformed into E. coli. The agarose gel electrophoresis confirms the presence of the C6 gene with the additional size of the restriction site and extra-base pairs used for restriction digestion. C6: positive control, lane 1–8: different E. coli clones. The vector map of pcDNA3.1-C6 with the site was used for cloning. (b) The C6 gene was cloned into a pEYFP-N1 plasmid and transformed into E. coli. The agarose gel electrophoresis confirms positive E. coli colonies with the C6 gene. Lane 1–10 indicates the different E. coli colonies and C indicates the positive control of the C6 gene. The vector map of pEYFP-C6 was used for cloning and as an open reading frame. (c) The C6YFP gene was cloned into pcDNA3.1- plasmid and transformed into E. coli. The agarose gel electrophoresis confirms positive E. coli colonies with the C6 gene. Lane 1–6 indicate the different E. coli colonies. pEYFP-C6 vector was used as a positive control. The vector map of pcDNA3.1-C6YFP with the site was used for cloning and as an open reading frame. Supplementary Figure 3. C6-YFP expression in CHO cells. (a) The C6-YFP transfected CHO cells were analyzed by flow cytometry for their fluorescence intensity (YFP) and represented as histogram plots. Data in the inset represent the mean fluorescence intensity (MFI). (b) The bar diagram represents the integrated mean fluorescence intensity (iMFI) of the transfected cells, further, confirm the presence of C6. Data exp [file 12879_2020_5372_MOESM1_ESM.pdf]

Supplementary Figure 1

a

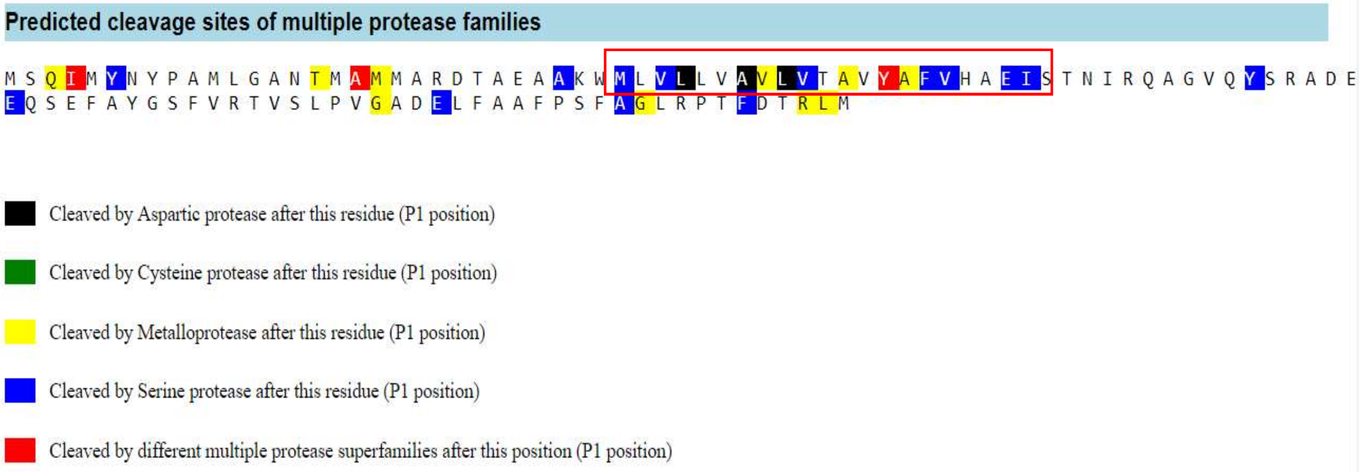

b

5’-GGATCC
ATGGCTACCGGCTCTCGGACCTCACTCCTCCTCGCTTTCGGACTGCTCTGTCTCCCTTGGCTCCAGGAAGGTTCTGCTTTCCCTACAATCCCCCTGAGCGAGATTTCCACTAAC
ATCCGGCAGGCTGGAGTGCAGTACTCTCGCGCAGACGAGGAACAGGCCGTGTATGCTTTTGTCGAGATTAGTACCAATATCCGACAGGCCGGGGTGCAGTACTCAAGGGC
TGATGAGGAACAGGCAGTGTATGCCTTCGTCCACATGCTGGTGCTGCTCGTGGCCGTCCTCGTGACCGCCGTGTACGCATTTGTGCACGCCGTCTATGCTTTCGTCCACATG
CTGGTGCTGTTAGTCGCCGTGCTCGTCACAGCAGTGTACGCCTTTGTGCACGCTGTCTATGCATTCGTGCATGCCAACACTATGGCCATGATGGCTAGAGACACCGCTGAGG
CCGCTAAGTGGGTCTACGCTTTTGTGCACGCAAATACAATGGCAATGATGGCCCGGGATACTGCAGAAGCAGCCAAATGGGTGTATGCTTTTGTGCACATGAGCCAGATTA
TGTACAACTATCCAGCCATGCTGGGTTATGCTTTCGTGCACATGTCCCAGATCATGTACAATTATCCCGCAATGCTGGGCTACGCCTTTGTGCACTCTGAGTTCGCCTATGGA
AGTTTTGTGCGCACAGTCTCACTGCCTGTGGGGGCTGACGAGGCAGTCTACGCCTTCGTGCACAGCGAATTTGCCTATGGTTCCTTCGTGAGAACCGTCTCTCTGCCAGTGG
GCGCTGACGAAGCAGTCTACGCCTTCGTGCATCTCTTCGCTGCATTTCCCTCCTTCGCCGGGCTGAGGCCTACCTTTGATACAAGACTCATGTACGCTTTCGTGCACCTGTTC
GCAGCCTTCCCTTCCTTCGCAGGACTCCGACCCACATTTGACACCAGACTGATG
TGAAAGCTT-3’

Supplementary Figure 2

a

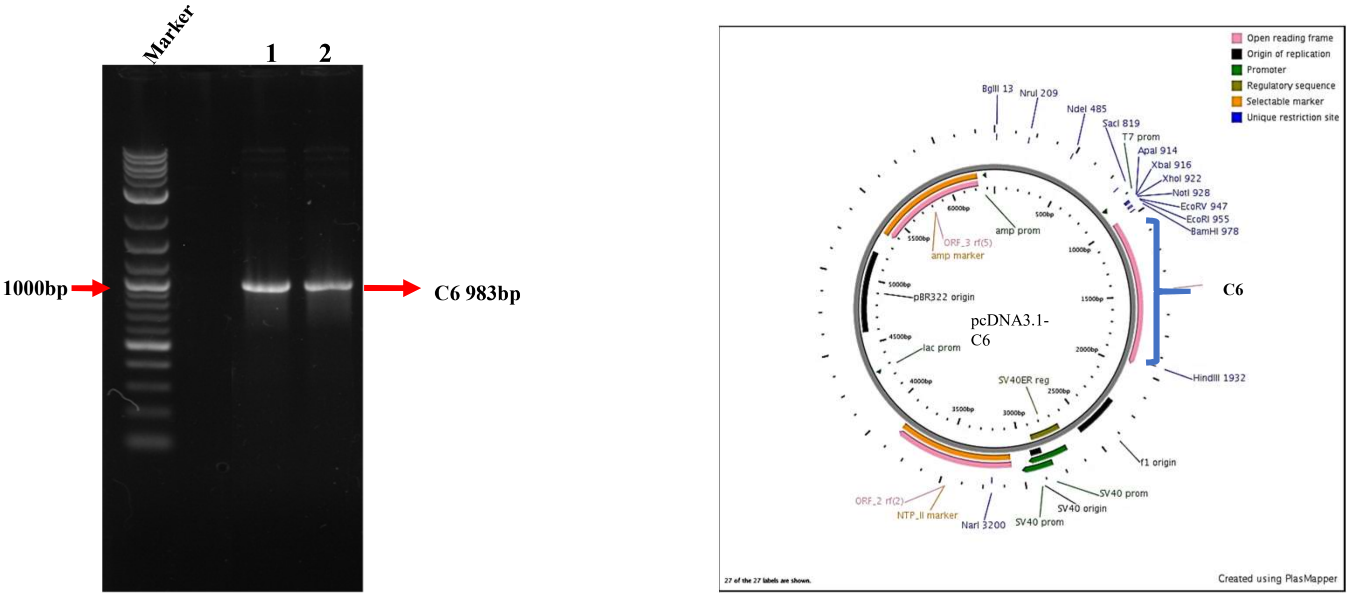

b

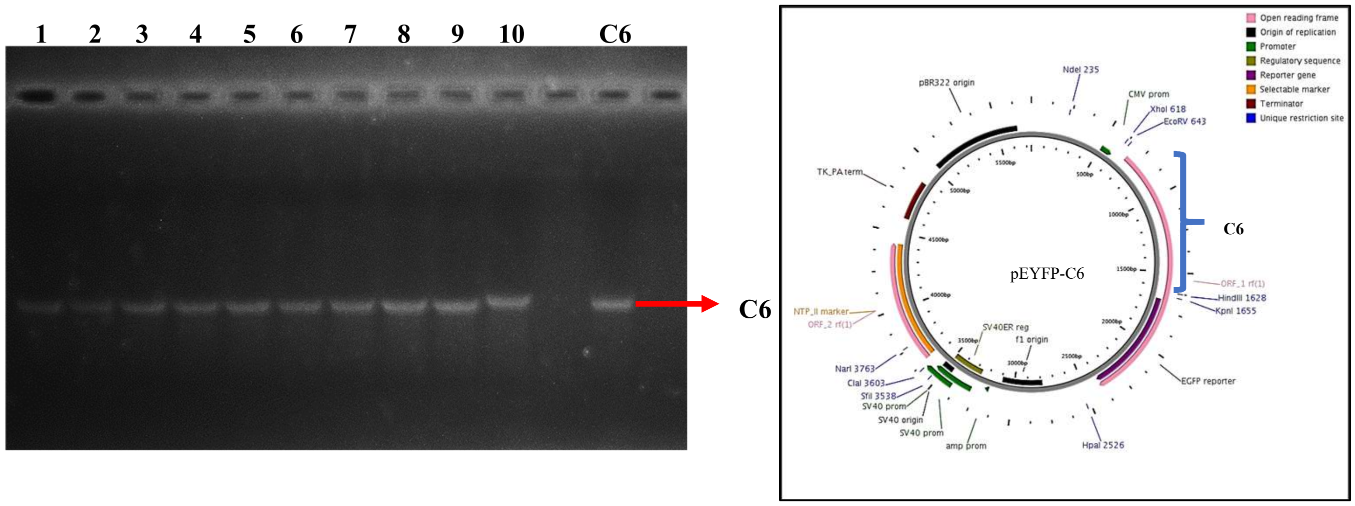

c

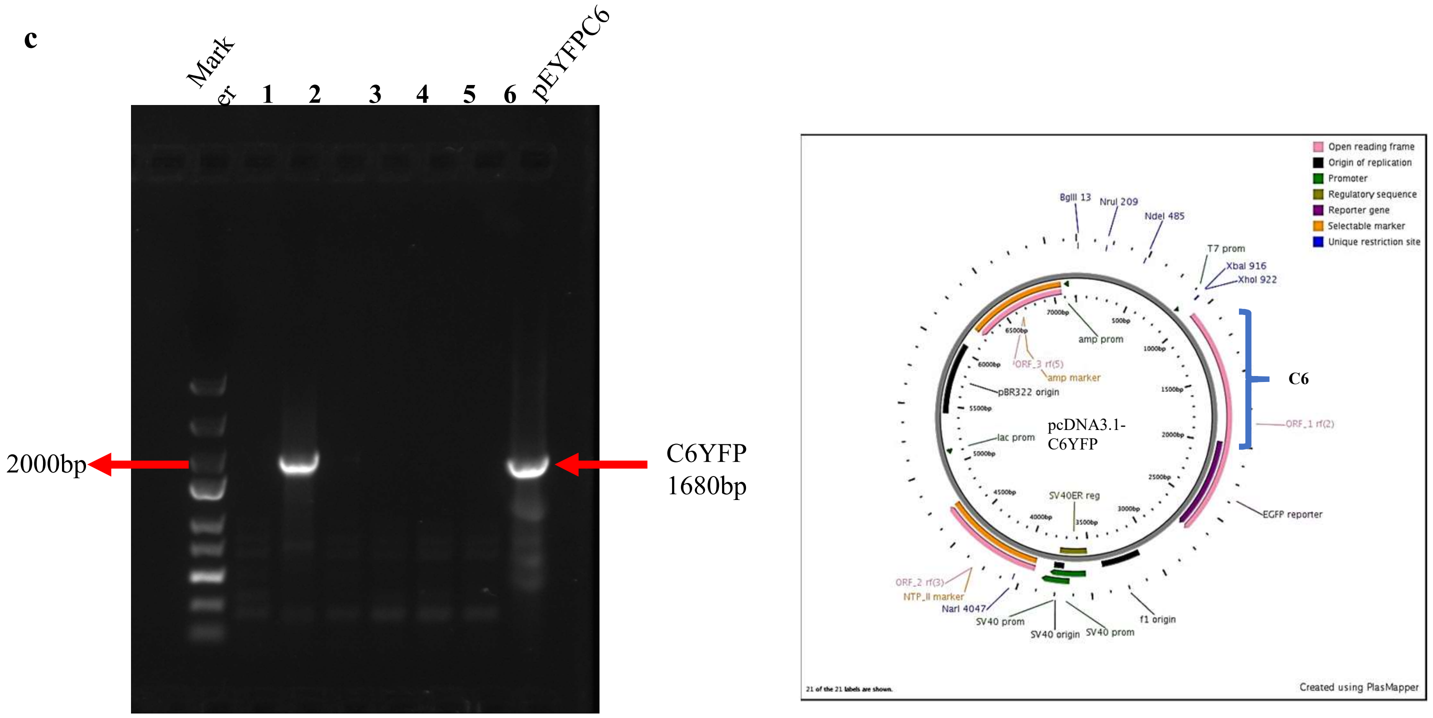

Supplementary Figure 3

a

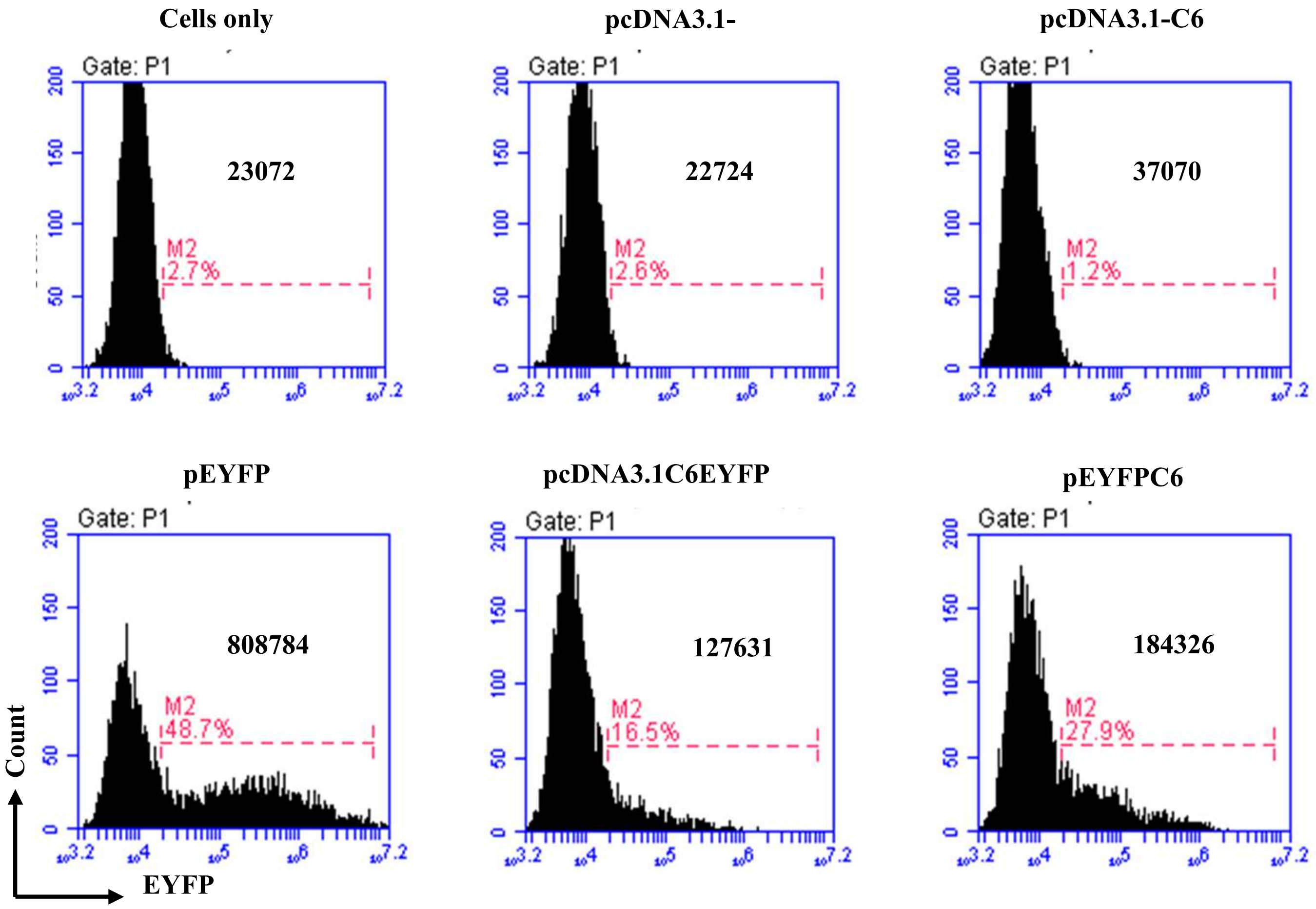

b

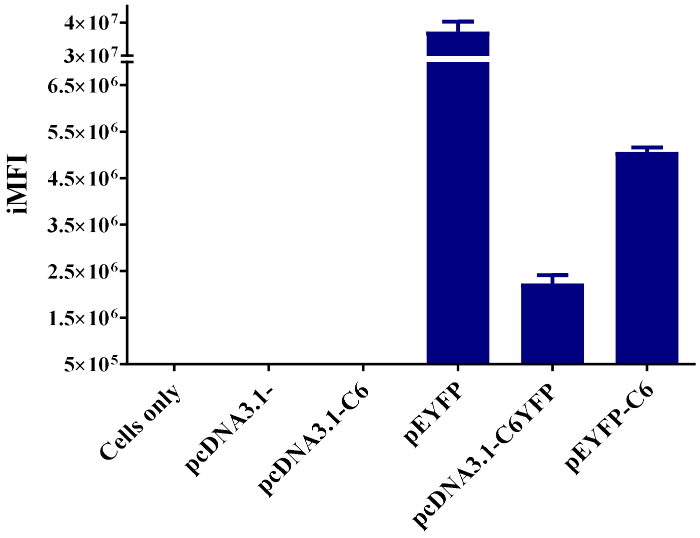

Supplementary Figure 4

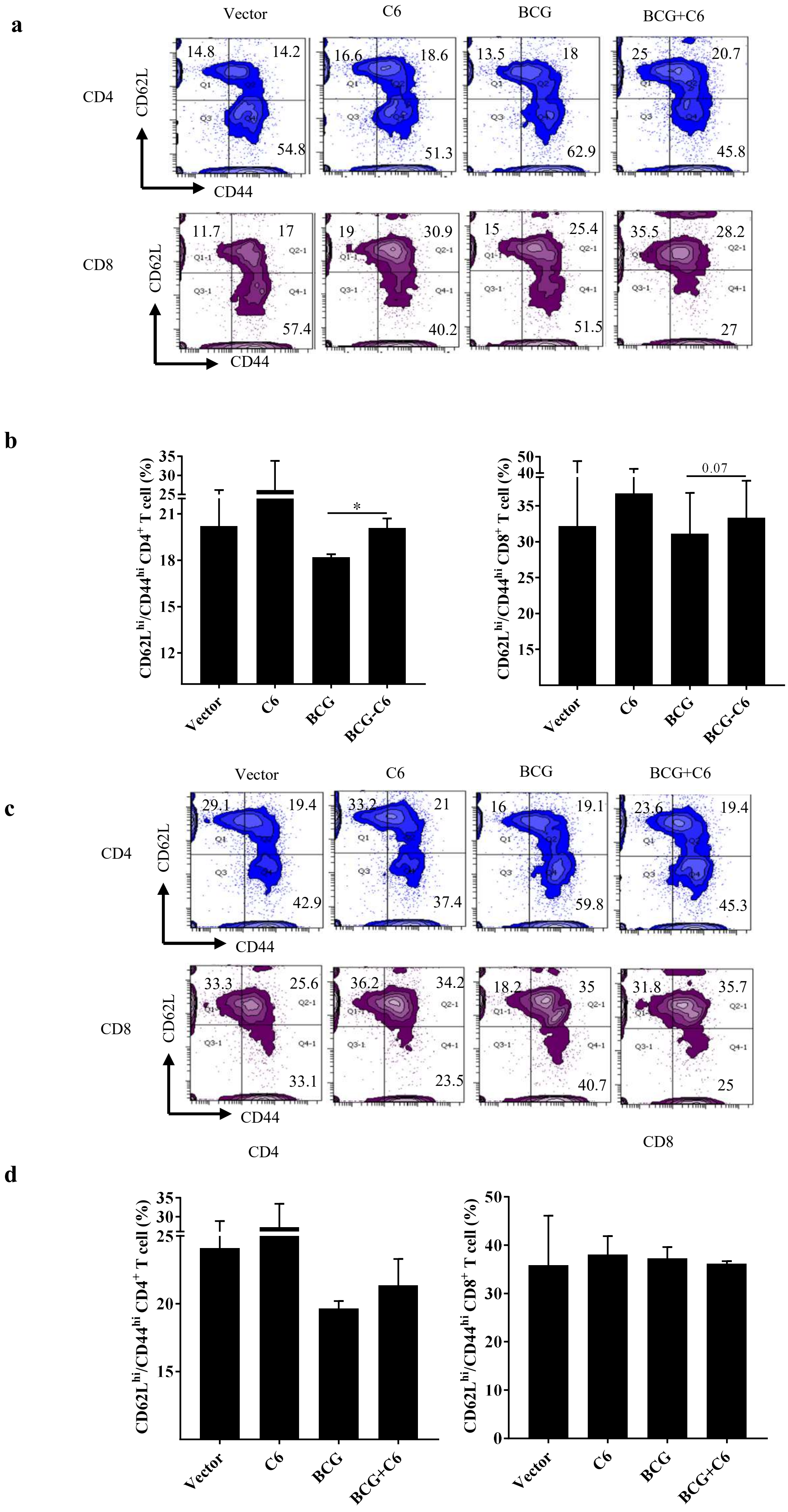

Supplementary Figure 5

a

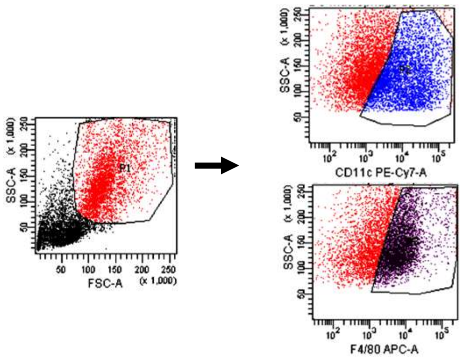

b

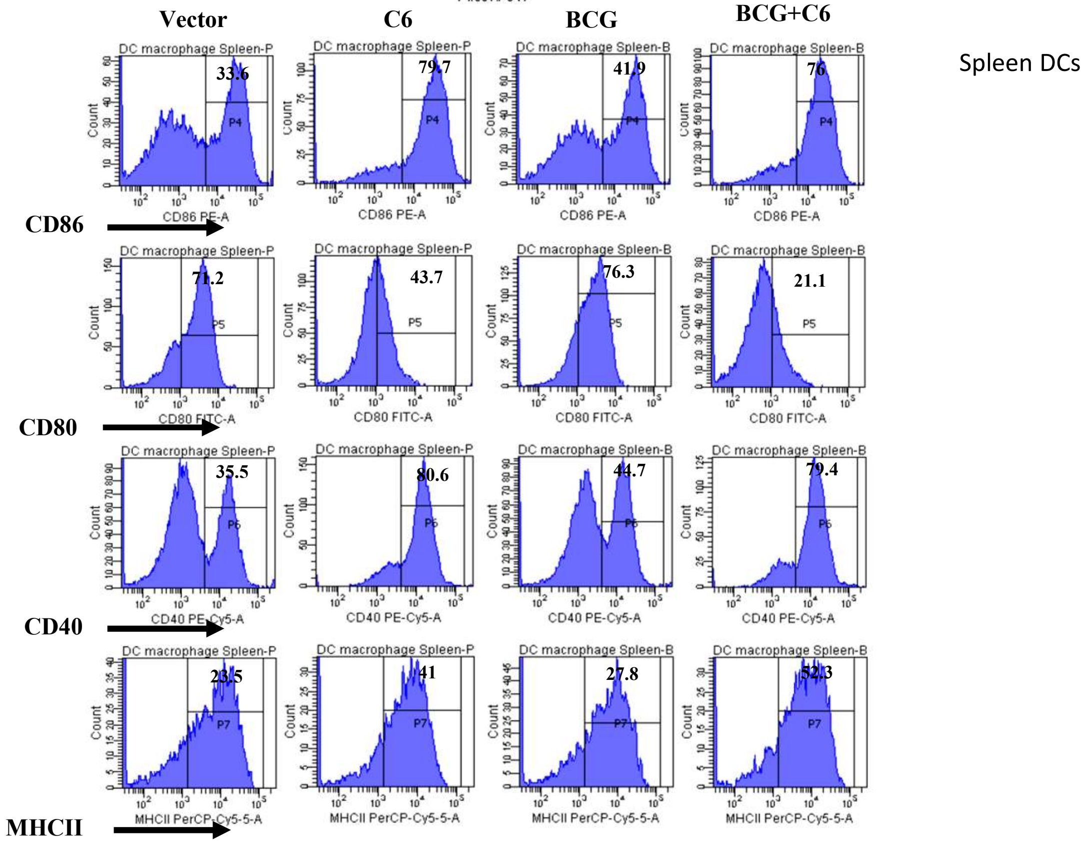

c

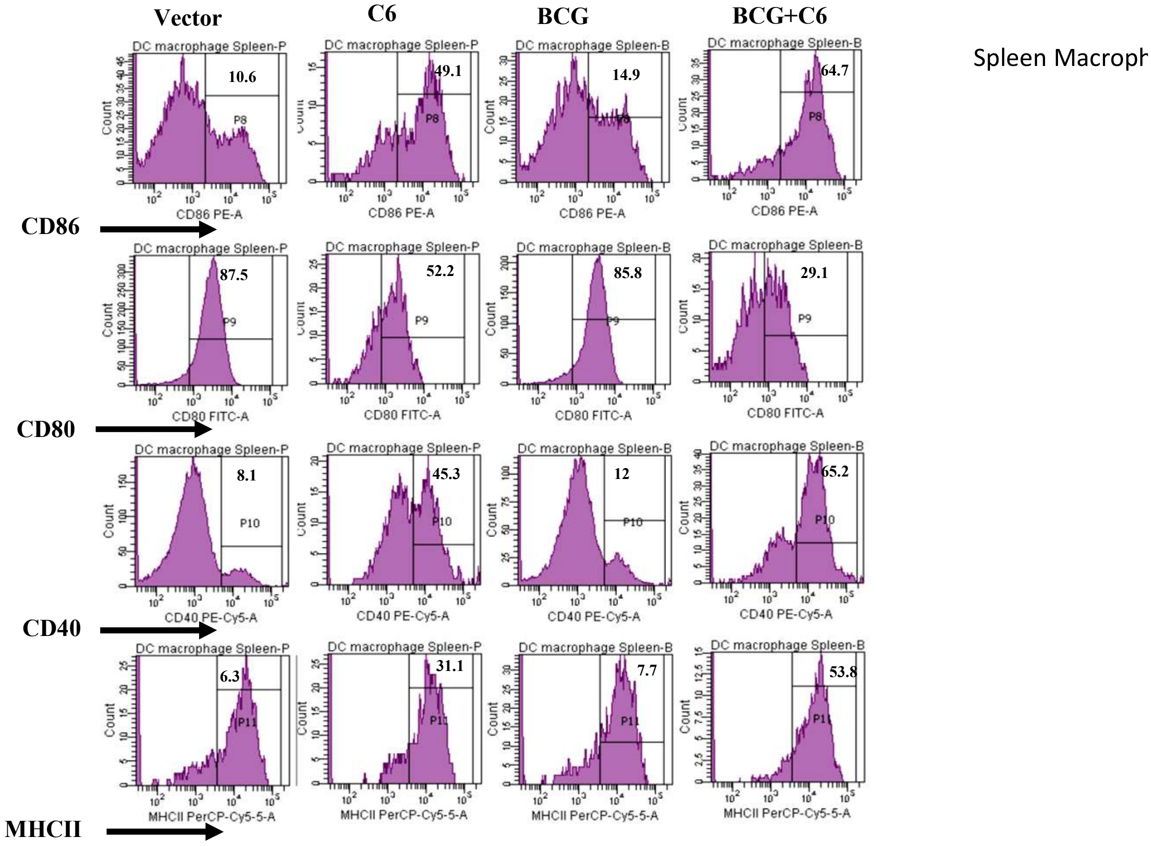

d

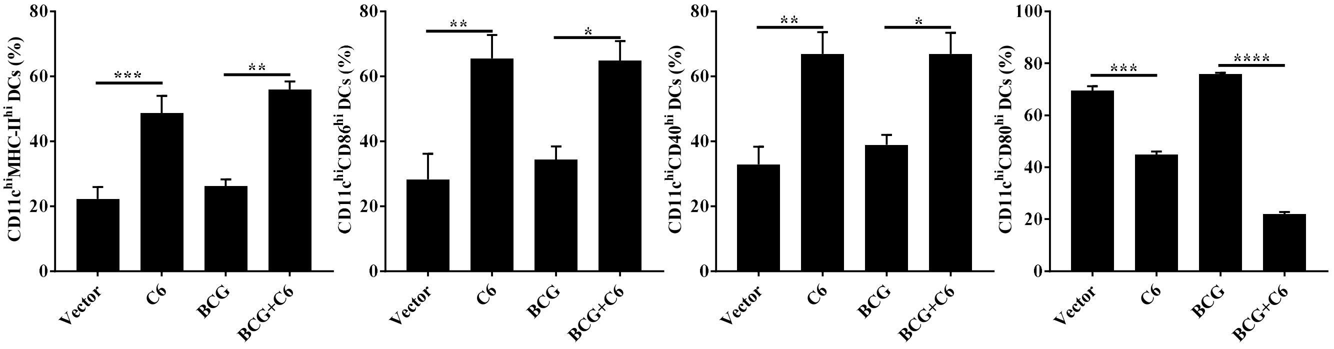
CD11c^{hi}MHC-II^{hi} DCs (%)
CD11c^{hi}CD86^{hi} DCs (%)
CD11c^{hi}CD40^{hi} DCs (%)
CD11c^{hi}CD80^{hi} DCs (%)
Vector
C6
BCG
BCG+C6
***
**
*
****

e

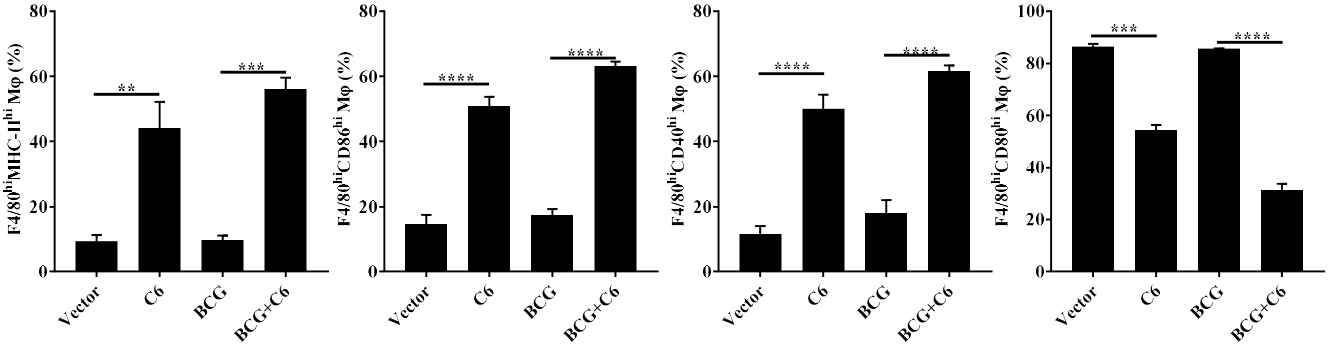
F4/80^{hi}MHC-II^{hi} Mφ (%)
F4/80^{hi}CD86^{hi} Mφ (%)
F4/80^{hi}CD40^{hi} Mφ (%)
F4/80^{hi}CD80^{hi} Mφ (%)
Vector
C6
BCG
BCG+C6
**
***
****
